# Excess Costs of Comorbidities in Chronic Obstructive Pulmonary Disease: A Systematic Review

**DOI:** 10.1371/journal.pone.0123292

**Published:** 2015-04-13

**Authors:** Manuel B. Huber, Margarethe E. Wacker, Claus F. Vogelmeier, Reiner Leidl

**Affiliations:** 1 Institute of Health Economics and Health Care Management, Helmholtz Zentrum München, German Research Center for Environmental Health (GmbH), Comprehensive Pneumology Center Munich (CPC-M), Member of the German Center for Lung Research (DZL), Neuherberg, Germany; 2 Department of Medicine, Pulmonary and Critical Care Medicine, Philipps-Universität Marburg, University Medical Centre Giessen and Marburg (UGMLC), Member of the German Center for Lung Research (DZL), Marburg, Germany; 3 Munich Center of Health Sciences, Ludwig-Maximilians-Universität, Munich, Germany; Pulmonary Research Institute at LungClinic Grosshansdorf, GERMANY

## Abstract

**Background:**

Chronic obstructive pulmonary disease (COPD) is a leading cause of morbidity and mortality worldwide. Comorbidities are often reported in patients with COPD and may influence the cost of care. Yet, the extent by which comorbidities affect costs remains to be determined.

**Objectives:**

To review, quantify and evaluate excess costs of comorbidities in COPD.

**Methods:**

Using a systematic review approach, Pubmed and Embase were searched for studies analyzing excess costs of comorbidities in COPD. Resulting studies were evaluated according to study characteristics, comorbidity measurement and cost indicators. Mark-up factors were calculated for respective excess costs. Furthermore, a checklist of quality criteria was applied.

**Results:**

Twelve studies were included. Nine evaluated comorbidity specific costs; three examined index-based results. Pneumonia, cardiovascular disease and diabetes were associated with the highest excess costs. The mark-up factors for respective excess costs ranged between 1.5 and 2.5 in the majority of cases. On average the factors constituted a doubling of respective costs in the comorbid case. The main cost driver, among all studies, was inpatient cost. Indirect costs were not accounted for by the majority of studies. Study heterogeneity was high.

**Conclusions:**

The reviewed studies clearly show that comorbidities are associated with significant excess costs in COPD. The inclusion of comorbid costs and effects in future health economic evaluations of preventive or therapeutic COPD interventions seems highly advisable.

## Background

Chronic obstructive pulmonary disease (COPD) causes around 5.6% of global deaths and presently constitutes the third leading cause of death, after stroke and ischemic heart disease, worldwide [1]. The persistent airflow limitation is associated with chronic inflammation in the airways, which is mediated by an increased expression of pro-inflammatory cytokines, chemokines, adhesion molecules, enzymes and receptors [2, 3]. The causes for COPD include environmental, as well as genetic factors. In developed countries the biggest risk factor for developing COPD is past or present smoking [4]. Around 15.4% of active smokers and 10.7% of ex-smokers are afflicted by COPD [5]. Not knowing if epithelial barrier dysfunction is cause or consequence of COPD, chemicals in tobacco smoke lead to down-regulation of tight junction genes [6] and promote dysregulation of the pulmonary epithelial barrier [7]. On the genetic side, alpha-1 antitrypsin (A1AT) deficiency is a significant risk factor but only accounts for around 2% of COPD cases [8]. The severity of COPD, among other factors, seems to correlate with a decreased diversity of the bronchial microbiome, as well as the presence of potentially pathogenic microorganisms and an increase of functions connected to pathogen-based inflammation [9–13]. The prevalence of multimorbidity among patients with COPD is significantly higher, than in patients without the disease [14–16]. Allocating causality between COPD and comorbidities is still difficult [17–19]. The reason for the increase and its influence on survival is not quite understood but in addition to shared risk factors like smoking and reduced physical activity, evidence is pointing towards a systemic inflammatory nature of COPD [20–24]. A shared component hypothesis, as proposed by network medicine, is currently evolving alongside technological progress [17, 25–28]. Aging [29] and increased survival into old age constitute congruent risk factors for developing COPD as well as comorbidities [30]. This co-occurrence generates significant costs but also offers reasonable leverage points to facilitate improved care, by either trying to prevent the development of specific comorbidities or by reducing their detrimental and often mutually reinforcing negative consequences. Strict study eligibility criteria often exclude COPD patients with comorbidities and therefore may fail to account for clinical reality [31]. The aim of this review is to accumulate latest evidence on the proportion and distribution of comorbid excess costs in COPD.

## Methods

### Definition of comorbidity

The debate about the definition of comorbidities is ongoing. One widely accepted position constitutes comorbidity as the occurrence of an index disease as well as at least one distinct additional entity in one person, while the term multimorbidity implies the occurrence of multiple acute or chronic diseases within one person but no index disease [32]. Some authors [33] require the index disease to cause the comorbidity and label diseases caused by perturbations of a shared cellular network or molecular pathway as multimorbidities. Cost results may thus depend upon whether or not comorbidities are required to interact with the index disease [34]. Yet, it may be difficult to specify, if a single disease is casually implicated in another disorder [17]. Comorbidity in this study therefore refers to the “classical” meaning in which COPD constitutes the index-disease and any additional disease affecting the same patient is labeled as comorbidity.

### Comorbidity assessment, costs and mark-up factors for COPD

Comorbidities can be assessed as single entity or as index. The index can either be quantitative e.g. patients are evaluated by the sole number of their comorbidities, or it can be weighted. In a weighted index, comorbidities with certain attributes e.g. a higher predictive mortality rate receive higher scores, whereas comorbidities with lower or no significant influence get reduced scores or are not considered at all. A widely used and well-accepted weighted tool is the Charlson Comorbidity Index (CCI) [35] as well as its ICD-9 based modification, the Charlson Deyo Index (CCI-Deyo) [36]. Conditions can receive scores of 1, 2, 3 or 6 and these are summed up to estimate mortality. Single assessment studies can also use indices to characterize and match study populations. In contrast to index-only studies however, they calculate the outcome for single comorbid conditions and therefore enable a comorbidity specific understanding of the respective cost influence.

This review only considered studies that directly stated or allowed the calculation of excess costs. Cost differences are expressed as mark-up factors which were calculated by dividing costs per patient in the comorbid case through costs per patient in the respective base case. Advantages of mark-up factors include their supplementary role alongside excess costs regarding the proportional change of the base case. For comparability, study costs were inflated by the national consumer price indices and converted to 2013 USD using gross-domestic product purchasing power parities [37–39]. Study quality was assessed independently by two reviewers using a criteria list derived from three assessment frameworks [40–42]. Possible study bias was reduced by separately stating comorbidity specific excess costs and by discussing respective results as well as limitations subsequently.

### Data sources, search strategy and study selection

Data extraction methods and the search strategy were conceptualized by two authors. A literature search was conducted by using the *PubMed* and Embase database. Results in languages other than English or German were excluded; a filter was set for journal articles. Reviews and conference abstracts were only used as supplementary information. The *PubMed* search included two separate passes. One was based on MeSH terms and the other was a standard free search with Boolean operators. MeSH-search terms included: (("Pulmonary Disease, Chronic Obstructive"[Mesh]) AND ("Costs and Cost Analysis"[Mesh] OR "Economics"[Mesh]) AND "Comorbidity"[Mesh]). 81 results were returned. The free search included: (COPD) AND (cost OR economic) AND comorbid* and resulted in 298 hits. The free-search included all results from the MeSH pass. An additional search was conducted by using Embase and applying the filter for journal articles. The search was based on the following subject headings connected by an “AND” operator: chronic obstructive lung disease, comorbidity, “health care cost”. 107 items were found.

### Data extraction

**Fig 1:**
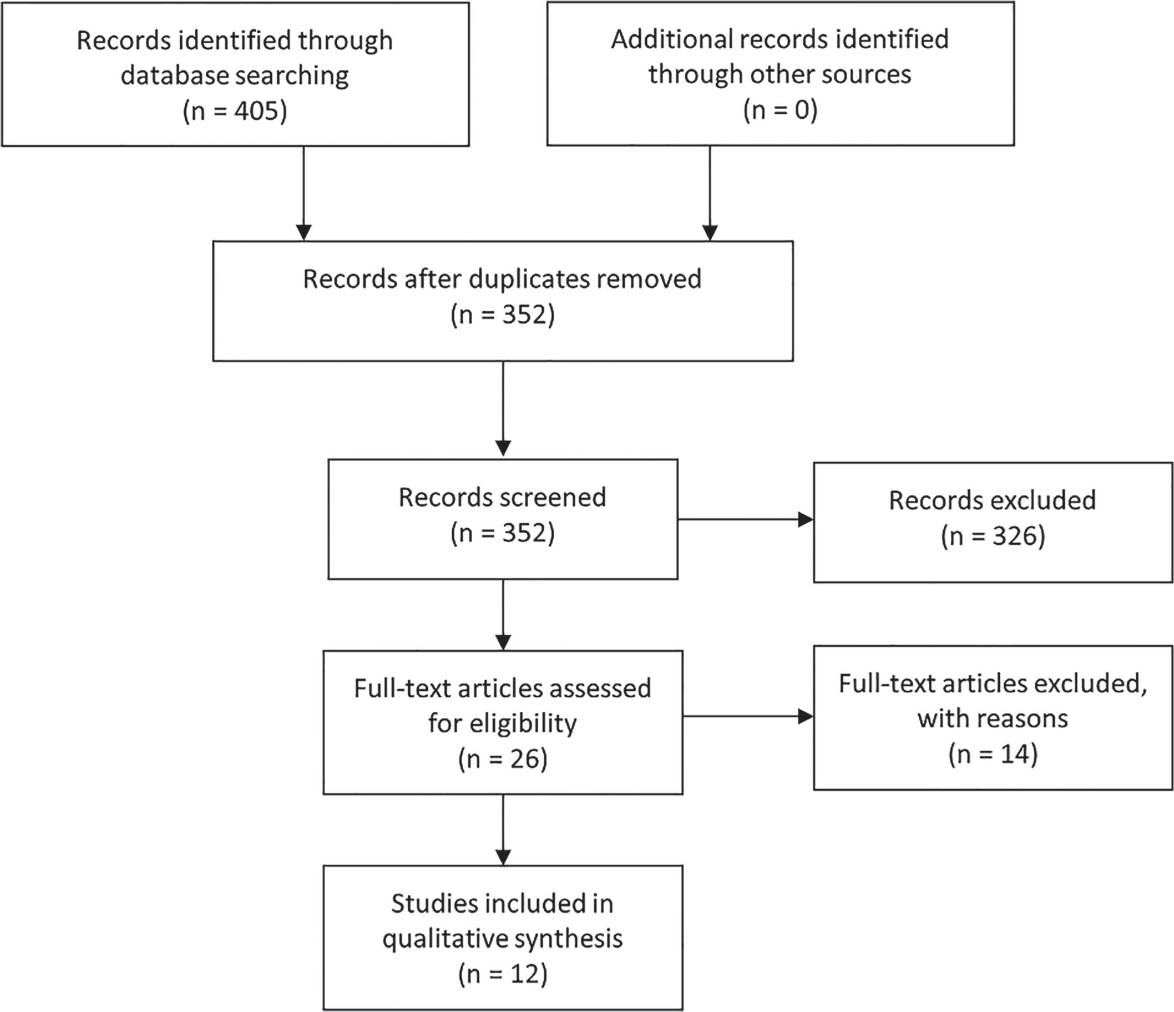
Literature search and study selection process.

After removing duplicates and studies which did not meet the language requirement, the results were screened, first by title, then by abstract. The abstracts were analyzed for keywords and content. The full text was acquired for studies deemed potentially relevant and a final decision regarding inclusion was made. The study selection process is illustrated by **Fig 1**, the PRISMA checklist can be found as S1 PRISMA Checklist. Due to a lack of costs, studies describing only healthcare utilization were excluded. While utilization is a good indicator, costs have the advantage of delivering a clear monetary picture of how comorbidities transform into economic burden. After removing duplicates, screening title plus abstract and conducting a full text analysis for the remaining results, twelve studies [43–54] remained. The selected terms of interest regarding data extraction were mainly based on well accepted items used throughout literature and other systematic reviews. Comorbid costs were the main focus of interest. Mark-up factors were calculated after the respective costs were extracted.

## Results

A summary of key study parameters, as well as results and mark-up factors is given in **Table 1**. Eight studies are located in the USA, and one each in Germany [51], Spain [54], Israel [53] and Italy [52]. Nine studies were published in 2009 or subsequently, the earlier ones are from 2006 [44], 2003 [52] and 2001 [50]. Most studies incorporate outcomes via comorbidity-specific assessment, only three incorporate outcomes based on binary presence or sole number of comorbidities [50, 52] or odds ratios (OR) for being in the upper 25, “most costly”, patient percentile [53]. The sample size differs significantly among studies and reaches from 99 patients with COPD and anemia [49] to 84,130 patients each for a group of COPD patients with or without pneumonia [45]. Five studies [44, 46, 48–50] used routine data located entirely or partly in the Medicare and Medicaid environment; the rest utilized routine or survey data from other sources. To control for differences between groups with and without comorbidity, three studies focused on pneumonia as comorbid disease [45, 47, 48], two studies focused on cardiovascular disease (CVD) [43, 54], one on anemia [44], one on sleep apnea syndrome [49] and two on multiple comorbidities [46, 51]. The rest focused on index-based outcomes. Four studies [43, 45, 47, 48] used Propensity Score Matching to adjust for patient heterogeneity; three studies [44, 53, 54] used multiple regression analysis and three studies [46, 49, 51] just matched for standard parameters including age, sex and comorbidities. The prevalent gender is female in half of the studies. The mean age is around or above 70 years in six studies [44, 45, 48, 50, 53, 54] and lower than 50 years in two [46, 47]. Three studies performed lung function tests to diagnose COPD and classify COPD severity by the Global Initiative for Chronic Obstructive Lung Disease (GOLD) definition [51, 53] or by the Spanish Society of Pneumology and Thoracic Surgery criteria in one study [54]. One study [52] used self-reported COPD diagnosis without severity classification. Due to a lack of COPD severity information in most of the utilized routine data, it could not be assessed or was not assessed in most of the studies. Two studies [43, 44] incorporated the utilization of oxygen therapy as indicator for severe cases of COPD.

**Table 1:** COPD study characteristics, cost categories, excess costs and respective mark-up factors.

**Study**	**Country**	**Gender (%)**			**Comorbidity of interest and evaluated cost category**	**Base case per patient and year**			**CD case per patient and year**				**Excess cost with CD** ^c^			**Mark-up factors**
	**Sample size**	**Mean age (± SD)**														
	**Source**	**Severity of COPD in %** ^a^														
	**Adjustment method**	**Prevalence of comorbidities** ^b^														
**Dalal et al. (2011) [43]**	- USA	- Male (C | C+CVD):		48.7 | 50.2	COPD + **Cardiovascular Disease**											
	- N(C) = 4,594	- Age (C):		63.8 ± 10.3	- All-cause medical:	$	8,695		$		24,621		$	15,926		(x 2.83)
	- N(C+CVD) = 4,594	- Age (C+CVD):		63.9 ± 10.0	- All-cause total:	$	12,450		$		29,249		$	16,799		(x 2.35)
	- IMS Lifelink database	- Oxygen therapy:		13.7% | 14%	- COPD-related medical:	$	1,147		$		2,046		$	899		(x 1.78)
	- PSM	- Mean CCI (C | C+CVD):		1.2 ± 1.3	- COPD-related total:	$	2,574		$		3,565		$	991		(x 1.39)
		- Mean CCI (C+CVD):		1.2 ± 1.5												
**Halpern et al. (2006) [44]**	- USA	- Male (C | C+A):		42.4 | 34.2	COPD + **Anemia**											
	- N(C) = 104,492	- Age(C):		74.7 ± 7.6	- Inpatient Claims^d^:	$	973		$		2,448		$	1,475		(x 2.52)
	- N(C+A) = 27,932	- Age(C+A):		77.5 ± 7.8	- Inpatient Payment^e^:	$	422		$		972		$	550		(x 2.30)
	- BEF	- Oxygen therapy (C | C+A):		3.7% | 9.8%	- Outpatient Claims:	$	178		$		280		$	102		(x 1.58)
	- Regression: demographic	- Circulatory disease (C | C+A):		26.0% | 26.8%	- Outpatient Payment:	$	44		$		77		$	33		(x 1.72)
	variables, COPD severity	- Endocrine + metab. (C | C+A):		7.9% | 8.5%	- Part B Claims:	$	414		$		824		$	410		(x 1.99)
		- Respiratory disease (C | C+A):		8.8% | 8.3%	- Part B Payment:	$	149		$		313		$	164		(x 2.10)
**Lin et al. (2014) [45]**	- USA	- Male (C | C+P):		50.9 | 51.2	COPD + **Pneumonia**											
	- N(C) = 84,130	- Age(C):		70.2 ± 12.3	- Inpatient:	$	4,332		$		20,459		$	16,127		(x 4.72)
	- N(C+P) = 84,130	- Age(C+P):		70.1 ± 12.5	- Outpatient:	$	8,565		$		16,307		$	7,742		(x 1.90)
	- CCE	- Mean CCI (C):		3.2 ± 2.3	- Prescription:	$	3,368		$		4,610		$	1,242		(x 1.37)
	- PSM	- Mean CCI (C+P):		± 2.6	- Total cost:	$	16,266		$		41,376		$	25,110		(x 2.54)
**Lin et al. (2010) [46]**	- USA	- Female (C | NoC):		78.2 | 78.2	Mean annual medical cost (p≤0.001)											
	- N(C) = 1,388	- Age (C):		± 6.5	- COPD + **CHF:**	$	14,066		$		18,919		$	4,853		(x 1.35)
	- N(Control) = 2,776	- Age (NoC):		± 6.6	- COPD + **Peptic ulcer:**	$	9,329		$		18,582		$	9,253		(x 1.99)
	- MMD	- Hypertension (C | NoC):		| 56.38	- COPD + **Liver disease:**	$	10,723		$		18,558		$	7,835		(x 1.73)
	- Regression: age (± 5	- Diabetes (C | NoC):		27.56	- COPD + **Diabetes:**	$	8,671		$		11,819		$	3,148		(x 1.36)
	years), sex, race	- CHF (C | NoC):		7.71	- COPD + **Diabetes** + **CC:**	$	8,492		$		18,534		$	10,042		(x 2.18)
		- Mean CCI-Deyo (C):		2.07	- COPD + **AIDS:**	$	12,413		$		17,041		$	4,628		(x 1.42)
		- Mean CCI-Deyo (NoC):		1.37 ± 2.03	- COPD + **Hypertension:**	$	8,029		$		10,387		$	2,358		(x 1.29)
**Menn et al. (2012) [51]**	- Germany	- Male (NoC | Stage I | Stage II):		47 | 55 | 60	Mean annual excess cost											
	-N (nC) = 1880	- Age (NoC):		± 12.7	- COPD + **Arthritis:**		n.a.				n.a.		$	663		n.a.
	- N (Stage I) = 267	- Age (Stage I):		± 13.5	- COPD + **Cancer:**		n.a.				n.a.			n.a.		n.a.
	- N (Stage II+) = 108	- Age (Stage II):		± 13.4	- COPD + **Diabetes:**		n.a.				n.a.			n.a.		n.a.
	- Kora-Age; Kora-F4	- Modified GOLD:			- COPD + **CHD:**		n.a.				n.a.			n.a.		n.a.
	- Regression: age, sex,	Stage I:		71%	- COPD + **Renal disease:**		n.a.				n.a.		$	4,819		n.a.
	education, smoking	Stage II+:		29%	- COPD + **Liver disease:**		n.a.				n.a.		$	6,137		n.a.
	status, comorbidity	- Arthritis (NoC | SI | SII+) in %:		| 11.2 | 13.9	- COPD + **Stroke:**		n.a.				n.a.			n.a.		n.a.
		- Cancer in %:		8.0 | 10.5 | 10.2												
		- Diabetes in %:		8.7 | 8.6 | 15.7												
**Miguel-Díez et al. (2010) [54]**	-Spain	- Male (C | C+CVD):		75.0 | 78.9	COPD + **Cardiovascular Disease**											
	- N(C) = 7,620	- Age (C):		65.92 ± 9.56	-Physician office visit:	$	203		$		240		$	37		(x 1.18)
	- N(C+CVD) = 1,770	- Age (C+CVD):		73.73 ± 8.29	- Specialist physician visit:	$	172		$		230		$	58		(x 1.33)
	- EPIDEPOC	- SEPAR criteria (C | C+CVD):			- Emergency department visit:	$	246		$		356		$	110		(x 1.45)
	- Regression: age, gender	Mild (FEV_1_: 60–80% ref.):		37.7 | 24.4	- Hospitalization:	$	1,272		$		2,887		$	1,615		(x 2.27)
		Moderate (FEV_1_: 40–59% ref.):		53.3 | 53.3	- Diagnostic tests:	$	238		$		319		$	81		(x 1.34)
		Serious (FEV_1_: <40% ref.):		8.9 | 22.3	- Drugs:	$	935		$		1113		$	178		(x 1.19)
		- Hypertension (C | C+CVD):		40.8% | 64.3%	- Oxygen therapy:	$	137		$		418		$	281		(x 3.04)
		- Hyperchol. (C | C+CVD):		37.7% | 44.5%	- Sick leave days:	$	149		$		73		$	76		(x 0.49)
		- Diabetes (C | C+CVD):		12.2% | 29.5%	- Total cost (incl. vaccination):	$	3,380		$		5,676		$	2,296		(x 1.68)
**Polsky et al. (2012) [47]**	- USA	- Male (NoP | P):		45.6 | 45.9	COPD + **Pneumonia**											
	- N(NoP) = 1,203,823	- Age(NoP):		46.5 ± 12.3	- Inpatient:	$	3,977		$		13,473		$	9,496		(x 3.39)
	- N(C) = 50,785	- Age(P):		46.8 ± 12.2	- Outpatient:	$	7,506		$		14,752		$	7,246		(x 1.97)
	- N(P) = 402,831	- Mean CCI-Deyo (NoP):		0.45 ± 1.02	- Pharmacy:	$	3,387		$		5,080		$	1,693		(x 1.50)
	- N(P+C) = 16,343	- Mean CCI-Deyo (P):		0.44 ± 1.07	- Absenteeism:	$	10,115		$		14,770		$	4,655		(x 1.46)
	- TR, CCE, HPM				- Short-term disability:	$	3,342		$		5,671		$	2,329		(x 1.70)
	- PSM				- Total medical:	$	14,869		$		33,305		$	18,436		(x 2.24)
					- Total productivity:	$	13,457		$		33,897		$	20,440		(x 1.52)
					- Total cost:	$	28,326		$		53,745		$	25,419		(x 1.90)
**Ryan et al. (2013) [48]**	-USA	**Pre PSM:**			COPD + **Pneumonia**											
	- N(P) = 9,984	- Female (NoC | C):		59.7 | 54.5	- Annual direct medical costs per											
	- N(C+P) = 9,984	- Age (NoC):		75.8 ± 7.3	patient^f^:	$	24,313		$		48,562		$	24,249		(x 2.00)
	- CCW	- Age (C):		77.4 ± 7.2												
	- PSM	- Diabetes (NoC | C):		21.0% | 33.6%												
		- CHF (NoC | C):		17.8% | 48.2%												
		- IHD (NoC | C):		35.5% | 65.4%												
		**Post PSM:** n.a.														
**Shaya et al. (2009) [49]**	- USA	- Female (C | C+SAS):		54.1 | 53.5	COPD + **Sleep Apnea Syndrome**											
	- N(C) = 3,356	- Age (C):		52.5 ± 6.7	- Inpatient:	$	5,475		$		10,062		$	4,587		(x 1.84)
	- N(C+SAS) = 99	- Age (C+SAS):		51.7 ± 6.0	- Outpatient:	$	140		$		785		$	645		(x 5.61)
	- MMD	- Mean CCI-Deyo (C | C+SAS):		3.6 ± 3.0	- Physician office visit:	$	534		$		682		$	148		(x 1.28)
	- Regression: age, sex, race, obesity, CCI, time	- Mean CCI-Deyo (C+SAS):		± 2.6	- Annual total cost:	$	6,148		$		11,529		$	5,381		(x 1.88)
**Studies not focusing on specific comorbidities**																
**Dal Negro et al. (2003) [52]**	- Italy		- Female (C):	30.5	COPD + **Comorbidity** (binary)											
	- N(C) = 400		- Age (C):	64.4 ± 10.9	- Mean societal cost^g^:	$	1,785		$		3,253		$	1,468		(x 1.82)
	- Confronting COPD Survey- N.a.		COPD severity (self-assessed) mild | moderate | severe in %:	31 | 55 | 12												
			- Patients with comorbidity:	40%												
			- Kidney disease:	19%												
			- Heart disease:	6%												
			- Hypertension:	6%												
**Simon-Tuval et al. (2011) [53]**	- Israel		- Male (“Most costly” | remainder):	84.7 | 75.3	COPD remainder vs. “**most costly**”											
	- N (Most costly) = 98		- Age (“Most costly” | remainder):	70 | 67	- Inpatient:	$	659		$		4,484			n.a.	n.a.	
	- N (Remainder) = 291		- FEV (% predicted):	44 | 49	- Surgeries:	$	291		$		2,777			n.a.	n.a.	
	- Clalit Health Services		- GOLD class 3 or 4:	62.2 | 51.2	- Diagnostic procedures:	$	388		$		734			n.a.	n.a.	
	- Regression: age, gender,		- Mean age-adjusted CCI-Deyo:	9 | 5	- Consultations:	$	189		$		302			n.a.	n.a.	
	BMI, COPD severity,				- Emergency Room Visit:	$	43		$		117			n.a.	n.a.	
	comorbidities, CCI,				- Medication:	$	469		$		2,015			n.a.	n.a.	
	smoking history, sleep quality				- Annual total cost:	$	2,062		$		10,512			n.a.	n.a.	
**Strassels et al. (2001) [50]**	- USA		- Male (C):	61	COPD + **Comorbiditiy**		**0 to 2 CDs** ^**h**^					**≥3 CDs**				
	- N (C) = 228		- Age (44 to 54; 55 to 64; 65 to 74	8.3% | 17.6% |	- Inpatient:	$	6,484			$		10,711				(x 1.65)
	- NMES		; ≥75):	43.9% | 30.3%	- Outpatient:	$	1,616			$		1,671				(x 1.03)
	- N.a.		- Arthritis:	59.5%	- Prescription:	$	826			$		1,271				(x 1.54)
			- Hypertension:	44.4%	- Physician office:	$	1,083			$		1,423				(x 1.31)
			- Heart disease:	29.8%	- Emergency department:	$	137			$		279				(x 2.04)
			- No. of CDs (0; 1; 2; ≥3) in %:	| 17 | 21 | 60	- Annual total cost:	$	10,146			$		15,356				(x 1.51)

All costs were inflated to 2013 USD by using consumer price indices from the OECD [37] (for costs in EUR) or the CPI inflation calculator [39] (for costs in USD). EUR were converted to USD by using gross-domestic purchase power parities [38] for 2013. If several years but no single price year was stated, the average year was used.

a: Oxygen therapy utilization as indicator for severity of COPD

b: Only comorbidities with the highest prevalence are shown

c: Annual excess cost per patient and category

d: Claims as submitted charges

e: Payments as actual reimbursements

f: Medicare and out-of-pocket but not outpatient pharmacy costs

g: Direct costs and absenteeism

h: Comorbidity groups were aggregated due to low population size

ref.: Of reference group; CD: Comorbid disease; PSM: Propensity Score Matching; C: COPD; CVD: Cardiovascular disease; A: Anemia; P: Pneumonia; SAS: Sleep apnea syndrome; NMES: National Medical Expenditure Survey; MMD: Maryland Medicaid Database; BEF: Beneficiary Encrypted Files; CCE: Commercial Claims and Encounters; TR: Thomson Reuters’ research proprietary databases; HPM: Health Productivity and Management database; CMS: The Centers for Medicare & Medicaid Services; CCW: Chronic Condition Warehouse via Centers for Medicare & Medicaid Services; CHF: Congestive heart failure; IHD: Ischemic Heart Disease; CC: Chronic complications; CHD: Coronary heart disease; n.a.: Not available

The existence of comorbid diseases was accounted for in every study either by assessing the CCI or CCI-Deyo score and/or by directly stating the prevalence of specific comorbidities in each group. The most prevalent comorbidities, in studies where this information was available, were circulatory diseases with a prevalence reaching from 25% to over 50% [44, 46, 48, 50, 54], as well as diabetes with a prevalence reaching from around 10% to 30% [44, 46, 48, 51, 54]. The CCI or CCI-Deyo scores differed significantly among studies and ranged from around 0.4 [47] to over 3 [45, 49, 53] in both groups respectively. Costs were reported in USD or EUR. The evaluated cost categories were partially identical among studies and had a clear focus on all-cause direct healthcare costs. Exceptions were the study by Dalal et al. 2011 [43], which distinguished between all-cause and COPD-related costs, as well as Polsky et al. 2012 [47], who incorporated indirect costs in the form of work absenteeism and short-term disability. Direct costs, if subdivided, consisted of inpatient costs, outpatient costs as well as prescription costs. This overall breakdown was done by seven studies [44, 45, 47, 49, 50, 53, 54]. Physician visits in studies from the United States resemble appointments outside of hospitals but not within hospital practices. The base case of COPD without comorbidity of interest differs significantly among studies. Four studies state figures from around 10,000 USD to 16,000 USD for total direct costs per patient and year [43, 45–47]. Halpern et al. 2006 [44] calculated a significantly smaller annual base case in the range of around 2,200 USD per patient. Three other studies [49, 53, 54] also calculated relatively low base cases. On the contrary, Polsky et al. 2013 [47] reached the highest base case of the studies under review, of around 28,300 USD. This high figure can partly be attributed to the inclusion of productivity losses. The ratio of inpatient to outpatient costs in the base case differs among studies. While inpatient costs are around 5 to 40 times higher than outpatient costs in the studies for anemia and Sleep Apnea Syndrome (SAS) [44, 49], the inpatient base costs for pneumonia are around half the size of outpatient base costs, in the respective studies for pneumonia [45, 47]. The prescription costs for pneumonia are around 3,400 USD per patient and year. Taking into account the comorbid case, a strong growth of inpatient costs can be observed. The respective mark-up factors increased to around 2.5 for anemia [44] and to around 3.4 [47] or 4.7 [45] in pneumonia, while the mark-up factors for outpatient costs increased to about 1.6, 2.0 and 1.9 respectively. In contrast to this development, the inpatient costs for SAS had a mark-up factor of 1.8, while outpatient costs more than quintupled in the same case [49]. Compared to the base case, comorbid direct costs increased among all studies. Renal and liver diseases created excess costs of 4,800 USD and 6,100 USD respectively [51]. The lowest increase of total excess costs for a single comorbidity could be seen for heart disease in Spain [54], comorbid anemia [44] as well as hypertension and diabetes without complications [46], while the highest increase of 25,110 USD [45] or 24,249 USD [48] or 25,419 USD [47] per patient and year could be observed for pneumonia.

An evaluation of basic quality criteria of all studies under review is illustrated in **Table 2**. The study perspective could be inferred or was directly stated in the majority of studies, other quality criteria like study limitations, source of funding and conflicts of interest were nearly always included. Funding through pharmaceutical companies was present in some studies. Parameters pertaining disease severity or prevalence of comorbidities or matching of patient groups were more heterogeneous. Inclusion criteria were stated in all studies; only Polsky et al. [47] focused on pneumonia patients and therefore did not state inclusion criteria for COPD patients. Assessment of study quality revealed significant heterogeneity in basic approaches and methods of analyzing comorbidity while transparency of reporting seemed adequate.

**Table 2:** List of quality criteria for comorbidity studies in COPD and their implementation in studies under review.

**Items** ^1^		**Studies**										
	Dalal et al. [43]	Halpern et al. [44]	Lin et al. [45]	Lin et al. [46]	Menn et al. [51]	Miguel-Díez et al. [54]	Polsky et al. [47]	Ryan et al. [48]	Shaya et al. [49]	Dal Negro et al. [52]	Simon-Tuval et al. [53]	Strassels et al. [50]
**Purpose of the study explained?**	✓	✓	✓	✓	✓	✓	✓	✓	✓	⊠	✓	✓
**Setting and location stated?**	✓	✓	✓	✓	✓	✓	✓	✓	✓	✓	✓	✓
**Study perspective stated directly or indirectly?**	✓	✓	✓	✓	✓	⊠	✓	⊠	✓	✓	✓	✓
**Epidemiological sources carefully described?**	✓	✓	✓	✓	✓	✓	✓	✓	✓	✓	✓	✓
**Were inclusion criteria for patient groups clear and sufficient?**	✓	✓	✓	✓	✓	✓	✓	✓	✓	✓	✓	✓
**Was the severity of COPD assessed or indicated?**	✓	✓	⊠	⊠	✓	✓	⊠	⊠	⊠	✓	✓	⊠
**Was an index used to indicate the prevalence and severity of comorbidities among groups?**	✓	⊠	✓	✓	⊠	⊠	✓	⊠	✓	⊠	✓	✓
**Were prevalences for single additional comorbidities stated?**	⊠	✓	⊠	✓	✓	✓	✓	✓	⊠	✓	⊠	✓
**Were comparison groups matched for characteristics?**	✓	⊠	✓	✓	⊠	⊠	✓	✓	⊠	⊠	✓	n.a.
**Did the outcome include direct costs?**	✓	✓	✓	✓	✓	✓	✓	✓	✓	✓	✓	✓
**Did the outcome include indirect costs?**	⊠	✓	⊠	⊠	⊠	✓	✓	⊠	⊠	✓	⊠	✓
**Was the price date stated?**	✓	✓	⊠	✓	✓	⊠	✓	✓	⊠	⊠	✓	✓
**Were study limitations stated?**	✓	✓	✓	✓	✓	✓	✓	✓	✓	⊠	✓	✓
**Was the source of funding stated?**	✓	✓	✓	✓	✓	✓	✓	⊠	⊠	⊠	✓	✓
**Were possible conflicts of interest stated?**	✓	✓	✓	⊠	⊠	✓	✓	✓	⊠	✓	⊠	✓

✓: Yes; ⊠: No; n.a.: not applicable; □: unknown

1: Items partly based on Husereau et al. [40], McKeage et al. [41], Molinier et al. [42]

## Discussion

**Fig 2:**
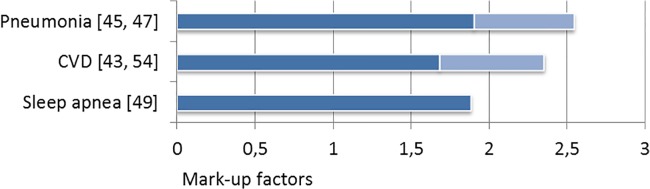
Comorbid mark-up factors for total costs.

**Fig 3:**
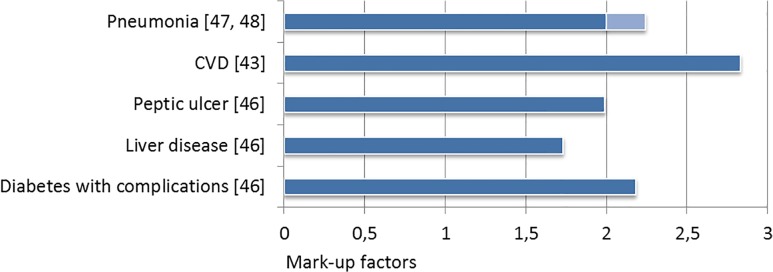
Comorbid mark-up factors for medical costs.

Among all studies analyzed in this review, comorbidities in COPD were associated with significant excess costs (**Figs 2 and 3**).

On average, the mark-up factors resemble a doubling of costs in the comorbid case. There were differences however, in the comorbidity-specific contributory extent. Pneumonia has been investigated most intensively and found to cause very high direct and indirect costs, not only in the base case, but especially as comorbidity in COPD. The pneumonia mark-up factors for total direct costs range between 1.9 and 2.5, while inpatient mark-ups peak at 3.39 and 4.72 respectively. In order to reduce excess costs, the implications of inhaled corticosteroid treatment for pneumonia risk in COPD patients [55, 56], the effect on inflammation markers [57, 58] as well as the usage in certain clinical COPD phenotypes [59] require further research. Clarification of when to apply inhaled-corticosteroids is still needed [60]. After pneumonia, CVD was connected to the second highest direct excess costs in one study [43]. Interestingly, age >65 years, severe COPD and comorbid Congestive Heart Failure (CHF) seem to also be associated with an increased risk for community-acquired pneumonia [61]. Beta blockers, one of the most important classes of drugs used to treat heart disease, may cause bronchoconstriction in patients with obstructive airway diseases [62]. Under-use of beta blockers in heart disease patients with COPD is documented [63]. However, looking at recent evidence, especially selective beta blockers show a good safety profile in patients with COPD and do not seem to be connected to increased all-cause mortality or exacerbations [64–68]. The GOLD document acknowledges these findings by stating that the potential benefits outweigh the risks [69]. From a differentiated perspective, this is one example where comorbidities and therapy are linked and interaction with cost is present. Total annual direct costs were 135% higher in patients with COPD and CVD, than in patients with COPD only, while COPD related costs were 38% higher in the concomitant group [43]. In the same study all-cause excess costs for comorbid CVD are around 16,800 USD per patient and year, while the mark-up factor reaches 2.35. Lin et al. 2010 [46] provide annual excess costs of around 4,900 USD for COPD and CHF which only transforms into a mark-up of 1.35. Contrary to these results, Miguel-Díez et al. [54] only reach excess costs of 2,300 USD in a Spanish setting. However, this still resembles a mark-up factor of 1.7. In another study [53] myocardial infarct and CHF increase the OR of being in the most costly quartile to around 6 (p < 0.001) and 7 (p = 0.001) respectively. In concordance, the co-existence of COPD and CVD increases the risk for hospitalization due to exacerbations and significantly reduces quality of life [70].

Evidence for the remaining comorbidities can only be drawn from a single study each. Depending on severity and presence of complications, the excess medical costs for comorbid diabetes were around 3,150 USD and 10,050 USD, the latter having a mark-up of 2.18 [46]. Unfortunately, despite its prevalence and importance [71], no additional studies stated numbers for diabetes. A smaller but also significant cost increase of around 2,700 USD excess costs can be seen for COPD and anemia. Mark-up factors for anemia reach from 2.30 to 2.52 for inpatient payments and inpatient claims respectively. An association between anemia and increased risk of mortality seems likely [44]. Peptic ulcer, liver disease and AIDS have mark-up factors of 1.99, 1.73 and 1.42 respectively [23]. In all three diseases the medical costs in the comorbid case are relatively high and range between around 17,000 USD and 18,500 USD. Sleep apnea had excess costs of 5,381 USD [49] and reached the highest mark-up factor among all studies and cost types of 5.61, for outpatient costs. Inpatient excess costs of 4,587 USD were still around 7 times higher, than outpatient excess costs in this case. Not surprisingly, the sole presence of one or more comorbidities is connected to higher excess costs [50, 52]. This is also true in the general context, where a clear association between number of chronic diseases and healthcare utilization as well as costs can be seen [72]. Interestingly, among all studies with available data, inpatient costs in the comorbid case were always the main cost driver. This finding is also confirmed by a recent study from Jansson et al. 2014 [73], who stated that by amounting to 46% of total costs, hospitalization due to comorbid conditions is the main cost driver among Swedish patients with COPD. Lowering inpatient utilization, by preventing possible drivers, like exacerbations in the case of COPD and CVD, should therefore constitute a main field of interest in order to reduce excess costs in these cases. Timely updated treatment guidelines may help to synchronize availability of latest evidence and their realization in the clinical practice. The GOLD-guidelines account for respective treatment implications by containing a full chapter on comorbidities in COPD [69]. Clear treatment advice is given for the comorbidity alongside COPD and regarding COPD alongside the comorbidity. We agree with Lehnert et al. [72, 74], who concluded that disease guidelines often fail to account for multimorbidity, and we would thus recommend the revision and updating of outdated disease guidelines regarding clear treatment advice in the presence of specific comorbidities.

Results for indirect costs were stated by three studies but differed significantly. Polsky et al. 2012 [47] stated that indirect costs were around 27% of total cost. Miguel-Díez et al. 2010 [54] only included sick leave days for indirect cost. As a consequence of this constraint and mostly retired study participants, indirect costs only were 1.3% of total costs in this case. The same reason could also apply to Dal Negro et al. 2003 [52], who stated indirect costs to be 3.6% of total costs. A recent review showed that with inclusion of morbidity or mortality costs, indirect costs constitute a substantial economic burden in COPD and range from 27% to 61% of total costs, depending on study population [75].

There were limitations of the studies under review. The overall study heterogeneity was high, not only because studies evaluated different comorbidities and different cost types but also because they used different data sources. Due to used routine data, the prevalent gender was female in half of the studies, despite the fact, that COPD universally is more prevalent in men [5]. The studies also stated different patient characteristics and failed to assess the severity of COPD and comorbidities in the majority of cases. Standardization of respective studies would enable better comparability. It became apparent, that the severity of comorbidities is currently mainly accounted for by using indices, which fail to address severity increments of several illnesses. Unless routine data starts to contain information about the severity of COPD and other diseases, cohort based study designs may have advantages in this regard. General limitations of the used routine data in the studies under review were misclassifications and non-generalizability of results due to predominance of specific populations e.g. low-income minorities [44–49, 53]. The implementation of standards pertaining baseline characteristics of patients as well as analysis seems warranted.

There are limitations of this review, too. In order to reduce heterogeneity, it was not actively searched for studies, which consider COPD as the comorbidity and other diseases as index disease. An in-depth comparison of costs among different countries and different healthcare systems is highly challenging [76]. Rather than detailing costing techniques, this study emphasized on the comparison of study approaches, offered conversion of cost results, and focused on the evaluation of excess costs and mark-up factors. Inferring whether these costs were justified or not, was not possible. Deducing the total economic burden per comorbid disease or drawing specific conclusions regarding the direct economic influence of COPD stage and comorbidity severity was not possible. Quality of life and mortality were not accounted for. It seems recommendable doing so in a further review since for example heart disease seems to have a strong detrimental influence on quality of life in COPD [77, 78]. A strength of this review is the aggregated tabular illustration of costs and mark-up factors for comorbidities in COPD. To our knowledge this has not been done before and helps to draw attention to economic leverage points of COPD.

From a health economic perspective the pressure for including comorbidities in economic evaluations of COPD interventions seems to mount. It is apparent, that due to complexity and heterogeneity of human disease and the real world setting it may be very difficult to transform the possible interconnectedness of disease into economic models. Therefore, evaluating the economic effect of comorbidity may still need to be handled irrespective of deciphering cause and effect or molecular connection. However, systems biology and network medicine are currently giving rise to a more advanced perspective of human disease, which may also enable more accurate health economic evaluations in the future. The human diseasome [79] (a platform linking disease phenotypic features with known disease genes) as well as its supplement, the interactome [25] (a platform linking disease with protein interactions) offer new ways to understand COPD and comorbidities. Both help to unravel and illustrate the interconnectedness of multimorbidities but challenge the current understanding of disease classification. Barbasi et al. [25] state that following recent evidence it would be difficult if not counter-intuitive, to consider human diseases as invariably independent. In concordance with this theory Grosdidier et al. [26] conclude that 16 “COPD multimorbidities” all share significant numbers of genes, proteins and biological pathways, which are targets of at least one chemical found in tobacco smoke. The current reductionist paradigm, where human disease and multimorbidity are viewed and classified as more or less isolated entities, does not account for an advanced and integrative shared component hypothesis [17, 25–28]. In addition to this, the very high failure rate of drug candidates in the prevailing “one disease, one target, one drug” paradigm seems to support this notion [80, 81]. The hurdles of regulation towards the co-development of drugs are still high but slowly accounted for by regulation authorities [82, 83]. Ignoring or not recognizing the economic implications of comorbidities in COPD will likely fail to target the true costs of the disease and the possible underlying disease network. In addition to this the overall effectiveness of respective multimorbid prevention and intervention methods will likely be underestimated.

## Conclusion

Acknowledging the accumulated data, comorbidities have a significant influence on care for COPD patients and subsequent excess costs. The available evidence is heterogeneous and far from comprehensive for all comorbidities of COPD, but delivers first insights regarding proportion and distribution of comorbid costs. Respectively, the calculated mark-up factors for different cost types range from 1.5 to 2.5 in the majority of cases and seem to double the costs on average. As presumed, there were significant differences in the comorbidity specific contributory extent. Comorbid pneumonia, CVD and diabetes with chronic complications were connected to relatively high excess costs, while comorbid anemia and arthritis were associated with relatively little cost influence. Main cost driver for comorbidities in all studies was inpatient cost. Indirect costs were not accounted for, by the majority of studies. Minimizing negligence of comorbidity associated treatment implications seems warranted and may be realized by adherence to timely updated treatment guidelines. Despite its inherent difficulty, the inclusion of comorbid influences on COPD should be promoted in future health economic evaluations of the disease.

## Supporting Information

S1 PRISMA Checklist PRISMA Checklist. (PDF)
